# CHA2DS2-VASc score in acute ischemic stroke with atrial fibrillation: results from the Clinical Research Collaboration for Stroke in Korea

**DOI:** 10.1038/s41598-020-80874-1

**Published:** 2021-01-12

**Authors:** Hak-Loh Lee, Joon-Tae Kim, Ji Sung Lee, Beom Joon Kim, Jong-Moo Park, Kyusik Kang, Soo Joo Lee, Jae Guk Kim, Jae-Kwan Cha, Dae-Hyun Kim, Tai Hwan Park, Sang-Soon Park, Kyung Bok Lee, Jun Lee, Keun-Sik Hong, Yong-Jin Cho, Hong-Kyun Park, Byung-Chul Lee, Kyung-Ho Yu, Mi Sun Oh, Dong-Eog Kim, Wi-Sun Ryu, Jay Chol Choi, Jee-Hyun Kwon, Wook-Joo Kim, Dong-Ick Shin, Sung Il Sohn, Jeong-Ho Hong, Man-Seok Park, Kang-Ho Choi, Ki-Hyun Cho, Juneyoung Lee, Hee-Joon Bae

**Affiliations:** 1 Department of Neurology, Chonnam National University Medical School, Chonnam National University Hospital, Gwangju, Korea; 2 Clinical Research Center, Asan Institute for Life Sciences, Asan Medical Center, University of Ulsan College of Medicine, Seoul, Korea; 3 Department of Neurology, Cerebrovascular Center, Seoul National University College of Medicine, Seoul National University Bundang Hospital, Seongnam-si, Korea; 4 Department of Neurology, Nowon Eulji Medical Center, Eulji University, Seoul, Korea; 5 Department of Neurology, Eulji University Hospital, Eulji University, Daejeon, Korea; 6 Department of Neurology, Dong-A University Hospital, Busan, Korea; 7 Department of Neurology, Seoul Medical Center, Seoul, Korea; 8 Department of Neurology, Soonchunhyang University Hospital, Seoul, Korea; 9 Department of Neurology, Yeungnam University Hospital, Daegu, Korea; 10 Department of Neurology, Ilsan Paik Hospital, Inje University, Goyang, Korea; 11 Department of Neurology, Hallym University Sacred Heart Hospital, Anyang, Korea; 12 Department of Neurology, Dongguk University Ilsan Hospital, Goyang, Korea; 13 Department of Neurology, Jeju National University Hospital, Jeju National University School of Medicine, Jeju, Korea; 14 Department of Neurology, Ulsan University College of Medicine, Ulsan, Korea; 15 Department of Neurology, Chungbuk National University Hospital, Cheongju, Korea; 16 Department of Neurology, Keimyung University Dongsan Medical Center, Daegu, Korea; 17 Department of Biostatistics, Korea University College of Medicine, Seoul, Korea

**Keywords:** Cardiology, Neurology

## Abstract

We investigated a multicenter registry to identify estimated event rates according to CHA2DS2-VASc scores in patients with acute ischemic stroke (AIS) and atrial fibrillation (AF). The additional effectiveness of antiplatelets (APs) plus oral anticoagulants (OACs) compared with OACs alone considering the CHA2DS2-VASc scores was also explored. This study retrospectively analyzed a multicenter stroke registry between Jan 2011 and Nov 2017, identifying patients with acute ischemic stroke with AF. The primary outcome event was a composite of recurrent stroke, myocardial infarction, and all-cause mortality within 1 year. A total of 7395 patients (age, 73 ± 10 years; men, 54.2%) were analyzed. The primary outcome events at one year ranged from 5.99% (95% CI 3.21–8.77) for a CHA2DS2-VASc score of 0 points to 30.45% (95% CI 24.93–35.97) for 7 or more points. After adjustments for covariates, 1-point increases in the CHA2DS2-VASc score consistently increased the risk of primary outcome events (aHR 1.10 [1.06–1.15]) at 1-year. Among OAC-treated patients at discharge (n = 5500), those treated with OAC + AP (vs. OAC alone) were more likely to experience vascular events, though among patients with a CHA2DS2-VASc score of 5 or higher, the risk of primary outcome in the OAC + AP group was comparable to that in the OAC alone group (P_int_ = 0.01). Our study found that there were significant associations of increasing CHA2DS2-VASc scores with the increasing risk of vascular events at 1-year in AIS with AF. Further study would be warranted.

## Introduction

Current practice guidelines recommend risk stratification with the CHA2DS2-VASc score to identify appropriate candidates for anticoagulation to prevent thromboembolic stroke in patients with atrial fibrillation (AF)^1^. Given that the CHADS2 scores omitted many potential risk factors and classified a large proportion of patients as being at ‘intermediate risk’^2^, the CHA2DS2-VASc scores have been proposed and validated to more accurately identify patients at truly low risk^3^. However, after acute ischemic stroke (AIS) in patients with AF, anticoagulation would be strongly indicated as stroke would score at least 2 on the CHA2DS2-VASc^4^. Moreover, in a meta-analysis and systematic review, anticoagulant therapy was superior to antiplatelet (AP) therapy for the prevention of stroke in patients with AF and recent nondisabling stroke or transient ischemic attack (TIA)^5^. Therefore, risk stratification concepts using the CHA2DS2-VASc score in patients with AIS and AF may need to be applied differently compared with that in patients with AF alone. For patients with AIS and AF, the CHA2DS2-VASc scores might be useful for estimation of the vascular event risks, not identification of appropriate candidates for OAC.

Most of the individual components of the CHA2DS2-VASc scores have previously been shown to be associated with stroke prognosis^6^. In addition, AF and atherothrombotic diseases share several risk factors and often coexist in the same patient^7,8^. High CHADS2 scores were related to a higher risk of atherothrombotic disease. Similarly, given the common coexistence of AF and atherothrombotic risks, it remains to be investigated whether OAC-only treatment could be optimal management in stroke patients with AF, especially those with high CHA2DS2-VASc scores.

We therefore investigated a multicenter registry to identify the estimated vascular event rates according to the CHA2DS2-VASc scores in patients with AIS and AF and the additional effectiveness of combined treatment with AP and OAC therapy compared with OAC alone, considering the CHA2DS2-VASc scores.

## Methods

### Subjects

This study is an analysis of a nationwide prospective, multicenter, stroke registry (Clinical Research Collaboration for Stroke in Korea, CRCS-K) database, which is a web-based database of consecutive patients with AIS or TIA admitted to 16 academic hospitals in South Korea. Detailed information about the registry has been reported previously^9,10^. From the CRCS-K registry database, we identified patients with AIS or TIA with AF who were hospitalized between January 2011 and November 2017. The inclusion criteria were as follows: 1) patients with AIS or neuroimaging-positive TIA hospitalized within 48 h of onset, 2) those with known AF or whose AF was diagnosed during hospitalization, and 3) those with mild-to-moderate stroke (National Institutes of Health Stroke Scale (NIHSS) scores ≤ 15). A detailed patient selection flowchart is presented in Supplemental Figure I.

### Ethics and data availability statement

The collection of clinical information in the CRCS-K registry was designed for the purpose of improving the quality of stroke care. The current study was approved by local institutional review boards of Seoul National University Bundang Hospital and Chonnam National University Hospital. The requirement for written informed consent from the study subjects was waived based on maintenance of study subject anonymity and minimal risk to the participants by institutional review boards in Seoul National University Bundang Hospital and Chonnam National University Hospital. All methods were performed in accordance with the relevant guidelines and regulations. The use of the registry database and additional review of the patients’ medical records and images in this study were also approved. Data used in this study are available upon reasonable request following submission of a legitimate academic research proposal to be assessed by the CRCS-K steering committee.

### Data collection

Demographic, clinical, imaging, and laboratory data were prospectively collected as previously reported^9,10^. The details are provided in the Supplemental Methods. We considered two different CHA2DS2-VASc scores: the CHA2DS2-VASc score, which did not add 2 points for the index stroke, and the poststroke CHA2DS2-VASc score, which considered 2 points for the index stroke if there was no previous stroke or TIA. The CHA2DS2-VASc score was categorized further as low (0–2), intermediate (3–4), and high (5 or more) scores considering the similar proportions of the population. Antithrombotic regimens at discharge were categorized into no OAC (no antithrombotics or AP only) and OAC with or without AP (OAC alone and OAC + AP).

### Outcomes

The primary outcome event was a composite of recurrent stroke (ischemic or hemorrhagic), myocardial infarction (MI), and all-cause mortality within 1 year of the index stroke. The secondary outcomes were (1) recurrent stroke (ischemic or hemorrhagic) and (2) all-cause mortality. Another outcome of interest was hemorrhagic stroke. Detailed definitions of the outcome events used in the current study were previously reported.^11^.

### Statistical analysis

The detailed methods for the statistical analysis are described in the Supplemental Methods. Briefly, we estimated event rates for primary vascular event outcome, recurrent stroke, and all-cause mortality for the individual CHA2DS2-VASc scores or poststroke CHA2DS2-VASc score in all patients, the OAC group (treated with OAC at discharge) and the no OAC group (not treated with OAC at discharge) by using the Kaplan–Meier method. We also investigated the risk of primary vascular event outcome by using Cox proportional hazard regression models. Adjustment variables were predetermined based on the clinical relevance as follows: age, initial NIHSS scores, and prior anticoagulation.

In addition, we performed weighted Cox proportional hazards regression analysis with rigorous adjustments for imbalances between the OAC + AP and OAC alone groups using inverse probability of treatment weighting (IPTW) and robust standard errors. We applied the cohort propensity score (PS) method in subgroup analyses^11^. Predetermined subgroups of the CHA2DS2-VASc score (low/intermediate/high score subgroups) were analyzed to determine the effectiveness of the addition of AP to OAC. To evaluate the effects of OAC + AP versus OAC alone and other covariates on outcomes, hazards ratios (HRs) and their 95% confidence intervals (CIs) were estimated using Cox regression models. Statistical significance was determined with a 2-tailed *P* value < 0.05. For the interaction analysis, considering the known insensitivity of interaction testing, heterogeneity was considered present with a *P* value < 0.10. All analyses were performed using SAS version 9.4 (SAS Institute Inc., Cary, NC).

### Ethics approval

The current study was approved by local institutional review boards at all participating centers, including Seoul National University Bundang Hospital and Chonnam National University Hospital.

### Consent to participate

The requirement for written informed consent from the study subjects was waived based on maintenance of study subject anonymity and minimal risk to the participants by institutional review boards in Seoul National University Bundang Hospital and Chonnam National University Hospital.

## Results

### General characteristics

Among all patients with stroke registered in the CRCS-K database during the study period, 59,512 patients with AIS were screened. Following enrollment (Supplemental Fig. 1), 7395 patients (mean age, 73 ± 10 years; men, 54.2%) were ultimately selected based on the eligibility criteria and analyzed. The median NIHSS score was 5 (IQR 2–10). The median CHA2DS2-VASc score was 3 (IQR 2–5). Patients with low CHA2DS2-VASc scores (0–2) made up 29.2% of the group; those with intermediate scores (3–4), 43.3%; and those with high scores (5 or more), 27.4%. At discharge, OAC was prescribed to 74.4% of patients. The following parameters had missing data that were substituted using median values: creatinine (0.2% of the data were missing), body mass index (1.2%), initial random glucose (1.0%), platelet count (0.1%), prothrombin time (0.7%), first fasting low-density lipoprotein (LDL) cholesterol (4.1%), and SBP (0.1%).

Baseline characteristics for all patients, no OAC group (n = 1895, 25.6%) and OAC groups (n = 5500, 74.4%) are shown in Table 1. The no OAC group was more likely to have a history of hypertension, diabetes mellitus, and large artery steno-occlusion and to have higher initial NIHSS scores and CHA2DS2-VASc scores.

**Table 1:** General characteristics of subjects.

	All patients	No OAC	OAC	P-value
N	7395	1895	5500	
Age, mean (SD)	73.1 (10.2)	74.3 (10.2)	72.7 (10.1)	< 0.001
Male, n (%)	4008 (54.2)	993 (52.4)	3015 (54.8)	0.07
**Arrival time, n (%)**				0.01
within 12 h	5968 (80.7)	1559 (82.3)	4409 (80.2)	
12–24 h	770 (10.4)	163 (8.6)	607 (11.0)	
24–48 h	657 (8.9)	173 (9.1)	484 (8.8)	
Prestroke mRS 0–1, n (%)	6296 (85.1)	1531 (80.8)	4765 (86.6)	< 0.001
BMI, mean (SD)	23.5 (3.5)	23.4 (3.4)	23.6 (3.5)	0.12
Baseline NIHSS, median (IQR)	5 (2—10)	8 (3—13)	5 (2—9)	< 0.001
**Risk factors, n (%)**				
Previous TIA	155 (2.1)	33 (1.7)	122 (2.2)	0.21
Previous stroke	1816 (24.6)	466 (24.6)	1350 (24.5)	0.97
Previous PAD	49 (0.7)	13 (0.7)	36 (0.7)	0.88
Previous CAD	1031 (13.9)	268 (14.1)	763 (13.9)	0.77
Hypertension	5209 (70.4)	1378 (72.7)	3831 (69.7)	0.01
Diabetes	2136 (28.9)	602 (31.8)	1534 (27.9)	0.001
Dyslipidemia	2030 (27.5)	433 (22.8)	1597 (29.0)	< 0.001
Smoking	1329 (18.0)	333 (17.6)	996 (18.1)	0.60
Congestive heart failure	319 (4.3)	77 (4.1)	242 (4.4)	0.53
**Medication history**				
Prior antiplatelet use	2668 (36.1)	723 (38.2)	1945 (35.4)	0.03
Prior anticoagulant use	1251 (16.9)	193 (10.2)	1058 (19.2)	< 0.001
Prior antihypertensive use	4472 (60.5)	1161 (61.3)	3311 (60.2)	0.41
Prior statin use	1694 (22.9)	378 (19.9)	1316 (23.9)	0.0004
Prior antidiabetic use	1617 (21.9)	470 (24.8)	1147 (20.9)	0.0003
**Laboratory findings, mean (SD)**				
WBC count, 10^3^/µL	8.12 (2.86)	8.62 (3.28)	7.94 (2.68)	< 0.001
Creatinine, mg/dl	1.05 (0.87)	1.08 (0.88)	1.04 (0.87)	0.09
Hemoglobin, g/dl	13.5 (2.0)	13.2 (2.1)	13.6 (1.9)	< 0.001
Platelet count, 10^3^/µL	207.2 (64.3)	206.8 (69.0)	207.3 (62.6)	0.78
Low-density lipoprotein, mg/dl	97.9 (32.3)	98.2 (32.9)	97.7 (32.1)	0.59
PT, INR	1.16 (0.40)	1.15 (0.42)	1.17 (0.40)	0.07
Glucose, mg/dl	138.5 (52.0)	143.7 (52.6)	136.7 (51.7)	< 0.001
SBP, mmHg	143.0 (25.8)	144.3 (27.6)	142.6 (25.2)	0.02
**LAD, n (%)**				< 0.001
no stenosis	3190 (43.1)	667 (35.2)	2523 (45.9)	
mild < 50%	368 (5.0)	77 (4.1)	291 (5.3)	
moderate > 50%	744 (10.1)	216 (11.4)	528 (9.6)	
occlusion	3093 (41.8)	935 (49.3)	2158 (39.2)	
Multiple lesions, n (%)	1255 (17.0)	384 (20.3)	871 (15.8)	< 0.001
**Recanalization therapy**				0.0001
no	5290 (71.5)	1281 (67.6)	4009 (72.9)	
IVT	1129 (15.3)	319 (16.8)	810 (14.7)	
IAT	470 (6.4)	142 (7.5)	328 (6.0)	
IV + IAT	506 (6.8)	153 (8.1)	353 (6.4)	
CHA2DS2-VASc, med (IQR)*	3 (2—5)	4 (2—5)	3 (2—5)	< 0.0001
**Stroke mechanism**				0.0001
Other than CE	1494 (20.2)	440 (23.2)	1054 (19.2)	
CE	5901 (79.8)	1455 (76.8)	4446 (80.8)	
**In-hospital treatment**				
Antidiabetics	1454 (19.7)	340 (17.9)	1114 (20.3)	0.03
Anti-HTN	3342 (45.2)	744 (39.3)	2598 (47.2)	< 0.001
Statin	5941 (80.3)	1250 (66.0)	4691 (85.3)	< 0.001

OAC, oral anticoagulant; mRS, modified Rankin Scale; BMI, body mass index; NIHSS, National Institutes of Health Stroke Scale; TIA, transient ischemic attack; PAD, peripheral arterial disease; CAD, coronary artery disease; WBC, white blood cell count; PT, prothrombin time; LAD, large artery disease; IVT, intravenous thrombolysis; IAT, intra-arterial therapy; CE, cardioembolism; HTN, hypertension.

### Outcomes according to the CHA2DS2-VASc scores

The median follow-up was 365 days (IQR 344–376). The primary composite outcome of recurrent stroke (ischemic or hemorrhagic), MI, and all-cause mortality occurred in 1294 patients, and the one-year cumulative event rate was 18.1% (95% CI 17.2–19.0). For individual outcomes, the one-year cumulative event rates were 5.6% (95% CI 5.1–6.2) for recurrent stroke, 0.59% (95% CI 0.40–0.78) for MI, and 14.6% (95% CI 13.8–15.4) for all-cause mortality.

Cumulative event rates of primary outcome events, recurrent stroke, and all-cause mortality at 1 year of follow-up according to CHA2DS2-VASc score are shown in Table 2 and Supplemental Table I. The primary outcome events at one year of follow-up ranged from 5.99% (95% CI 3.21–8.77) for a CHA2DS2-VASc score of 0 point to 30.45% (95% CI 24.93–35.97) for 7 or more points. The recurrent stroke event rates ranged from 1.43% for 0 points to 10.03% for 7 or more points, and all-cause mortality rates were 4.63% to 26.29%. For OAC-treated patients, the primary outcome events at one year of follow-up ranged from 2.05% (95% CI 0.03–4.07) for a CHA2DS2-VASc score of 0 point to 21.76% (95% CI 15.81–27.70) for 7 or more points. The recurrent stroke event rates ranged from 0.95% for 0 points to 7.51% for 7 or more points, and all-cause mortality rates were 1.12% to 18.16%. Adjusted cumulative event rates at 1 year according to the CHA2DS2-VASc scores are shown in Supplemental Table II. For all events, the unadjusted and adjusted event rates were substantially lower in the OAC group than in the no OAC group. Additionally, estimated event rates according to poststroke CHA2DS2-VASc scores (primary outcome events; 2.84% for 2 points to 19.63% for 8 or more points) are shown in Supplemental Table III.

**Table 2:** Cumulative event rates at 1 year (%) according to the CHA2DS2-VASc scores.

CHA2DS2-VASc score	All patients (N = 7395)		Non-anticoagulated patients (n = 1895)		Anticoagulated patients (n = 5500)	
	No. of events	Event Rate (%, 95% CI)^a^	No. of events	Event rate (%, 95% CI)^a^	No. of events	Event Rate (%, 95% CI)^a^
0	18	5.99 (3.21–8.77)	13	18.80 (9.59–28.02)	5	2.05 (0.03–4.07)
1	53	7.45 (5.49–9.41)	37	23.50 (16.75–30.24)	16	3.07 (1.57–4.56)
2	151	13.65 (11.59–15.72)	84	33.69 (27.72–39.66)	67	7.85 (6.00–9.70)
3	270	17.98 (16.01–19.95)	159	40.57 (35.63–45.52)	111	10.03 (8.23–11.83)
4	316	20.04 (18.02–22.07)	174	41.28 (36.49–46.07)	142	12.30 (10.34–14.25)
5	250	22.68 (20.15–25.21)	124	40.55 (34.92–46.19)	126	15.94 (13.33–18.56)
6	152	26.24 (22.55–29.94)	72	44.47 (36.67–52.26)	80	19.25 (15.30–23.21)
7 or more	84	30.45 (24.93–35.97)	43	50.63 (39.70–61.55)	41	21.76 (15.81–27.70)

All *P*-values < 0.001 by log-rank test.

Event: primary outcome.

**Figure 1:**
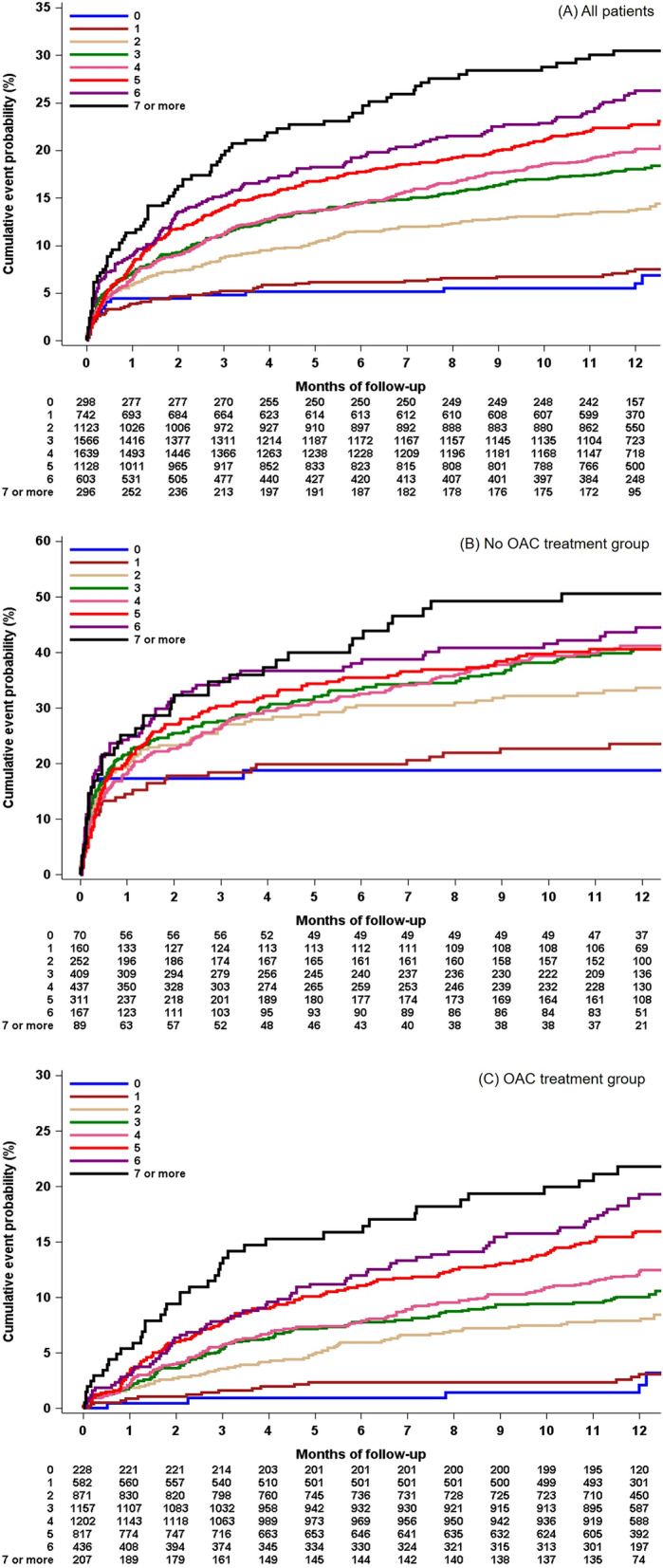
Kaplan–Meier curve of primary vascular outcome according to the individual CHA2DS2-VASc scores in the all-patient group (**A**), no OAC treatment group (**B**), and OAC treatment group (**C**).

After adjustments for covariates (model 1; age, initial NIHSS score, and prior anticoagulation), for the OAC-treated patients, 1-point increases in the CHA2DS2-VASc score consistently increased the risk of primary outcome events (adjusted HR 1.19 [1.12–1.25], *p* < 0.001), recurrent stroke (adjusted HR 1.29 [1.18–1.40], *p* < 0.001), and all-cause mortality (adjusted HR 1.19 [1.11–1.27], *p* < 0.001) at 1 year of follow-up (Table 3 and Supplemental Table IV). Additionally, compared with the lowest risk of 0 points for the CHA2DS2-VASc score, each higher point of the CHA2DS2-VASc score seemed to be associated with a greater risk of primary outcome events, all recurrent stroke, and all-cause mortality by unadjusted and adjusted analysis (Table 3). Supplemental Tables V and VI show the association of the CHA2DS2-VASc score with vascular outcomes at 1-year follow-up in all patients and the no OAC group. In all patients, 1-point increases in the CHA2DS2-VASc score significantly increased the risk of primary outcome events (aOR 1.10 [1.06–1.15]), recurrent stroke (aOR 1.25 [1.16–1.34]), and all-cause mortality (aOR 1.08 [1.04–1.13]), while among the groups treated with no OACs, the CHA2DS2-VASc scores were not associated with the risk of primary outcome events and all-cause mortality. Kaplan-Meier estimates of primary outcome, recurrent stroke, and all-cause mortality according to the CHA2DS2-VASc score are shown in Fig. 1.

**Table 3:** Association of CHA2DS2-VASc scores with primary outcomes in the OAC treatment group (n = 5500).

	Crude HR (95% CI)	*P*	Model 1 (HR, 95% CI)	*P*	Model 2 (HR, 95% CI)	P
Per 1-point increase	1.31 (1.25–1.38)	< 0.001	1.19 (1.12–1.25)	< 0.001	1.21 (1.14–1.28)	< 0.001
0	Ref		Ref		Ref	
1	1.28 (0.47–3.48)	0.63	1.06 (0.39–2.89)	0.91	1.17 (0.43–3.20)	0.76
2	3.60 (1.45–8.93)	0.01	2.22 (0.89–5.53)	0.09	2.57 (1.03–6.43)	0.04
3	4.58 (1.87–11.23)	0.001	2.36 (0.95–5.84)	0.06	2.79 (1.12–6.95)	0.03
4	5.66 (2.32–13.82)	0.0001	2.61 (1.05–6.45)	0.04	3.25 (1.30–8.09)	0.01
5	7.48 (3.06–18.29)	< 0.001	3.35 (1.35–8.30)	0.01	4.25 (1.70–10.64)	0.002
6	9.16 (3.71–22.62)	< 0.001	3.91 (1.56–9.82)	0.004	4.81 (1.90–12.18)	0.001
7 or more	10.45 (4.13–26.45)	< 0.001	4.08 (1.58–10.51)	0.004	5.34 (2.05–13.94)	0.001

Model 1: age, initial NIHSS, prior anticoagulation.

Mode 2: age, initial NIHSS, BMI, arrival time, dyslipidemia, smoking, prior anticoagulation, prior statin, antihypertensive treatment, antidiabetic treatment, statin treatment, reperfusion therapy, creatinine, glucose, SBP, large artery diseases, multiple lesions, stroke mechanism (TOAST).

### OAC vs OAC + AP

**Figure 2:**
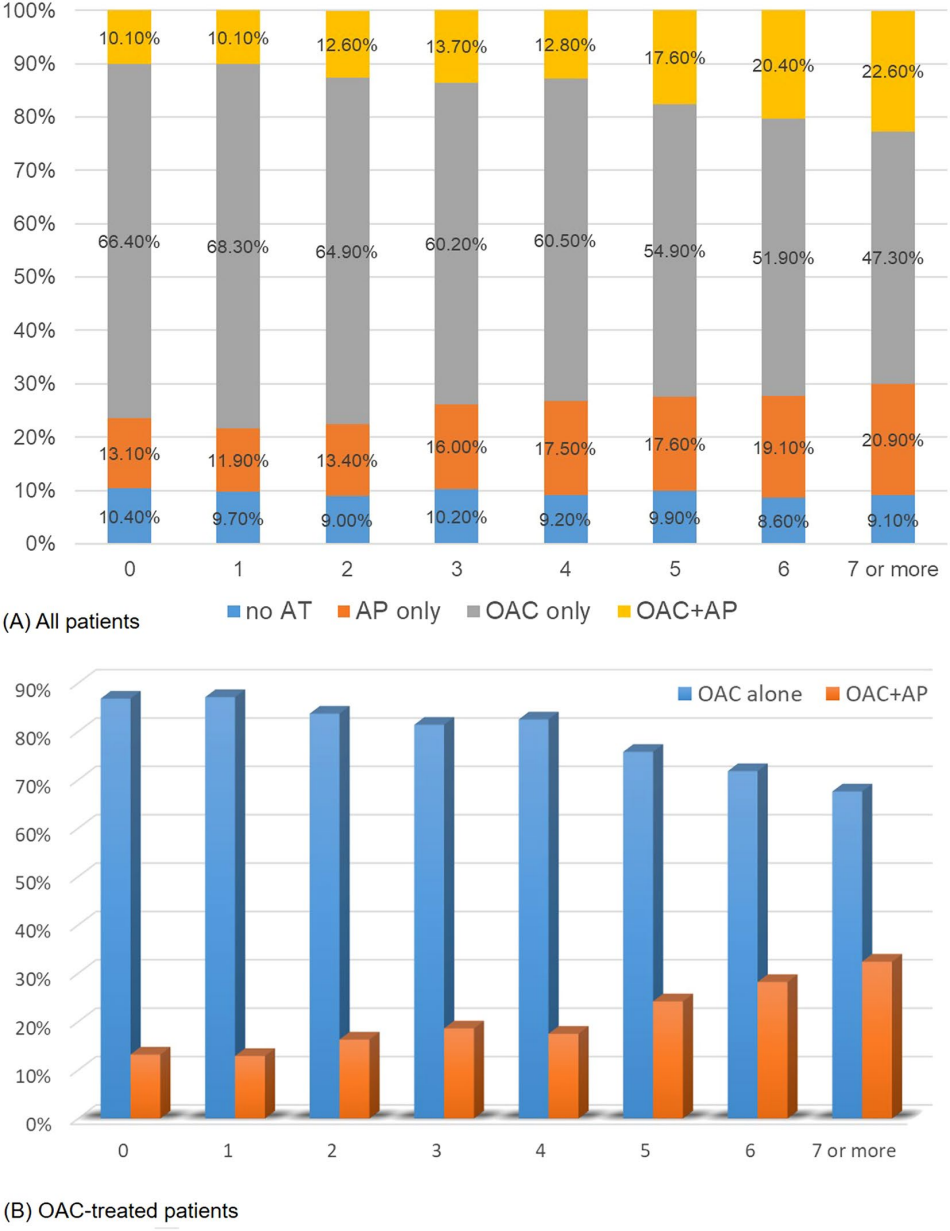
Proportions of antithrombotic treatment according to the CHA2DS2-VASc scores in all patients (**A**) and OAC-treated patients (**B**).

Among OAC-treated patients at discharge (n = 5500), the characteristics of the patients who received OAC alone (n = 4440, 80.7%) vs OAC + AP (n = 1060, 19.3%) are shown in Supplemental Table VII. Moreover, as CHA2DS2-VASc scores increased, the proportions of OAC + AP treatment increased up to 32% in patients with CHA2DS2-VASc scores of 7 or more (Fig. 2).

The OAC + AP group was more likely to have a history of stroke, peripheral artery disease, coronary artery disease, hypertension, diabetes mellitus, and smoking; have large artery diseases with moderate-to-severe stenosis; and be on antiplatelet agents, antihypertensives, antidiabetics, and statin medication at stroke onset than the OAC group. After IPTW, the distributions of the baseline characteristics were fairly well balanced (Supplemental Tables VIII).

The primary outcome events within one year occurred significantly more often in the OAC + AP group than in the OAC alone group (15.6% vs 10.1%, respectively, *p* < 0.001) (Supplemental Table IX). The 1-year recurrent stroke (7.0% vs 3.7%, respectively; *p* < 0.001) and all-cause mortality risks (11.3% vs 7.3%, respectively; *p* < 0.001) were significantly higher in the OAC + AP group than in the OAC alone group. In the PS analyses using the IPTW, primary outcome, all recurrent stroke, and all-cause mortality were significantly more frequent in the OAC + AP group than in the OAC group, with weighted absolute risk differences of 6.6%, 3.3%, and 5.6%, respectively (Supplemental Table IX).

### OAC versus OAC + AP according to the CHA2DS2-VASc score subgroups

Comparisons between the OAC alone and OAC + AP groups according to the CHA2DS2-VASc score subgroups are shown in Supplemental Table IX The crude and adjusted event rates of primary outcome events, recurrent stroke, and all-cause mortality were significantly higher in the OAC + AP group than in the OAC alone group among the low and intermediate score subgroups, while among the high score subgroup, the 1-year event rates were not significantly different between the OAC + AP group and the OAC group (Table 4 and Supplemental Table X). There was a potential interaction between the CHA2DS2-VASc subgroups and OAC at discharge (OAC and OAC + AP) on primary outcome events (P_interaction_ = 0.01). Briefly, among patients in the high score subgroup, the risk of primary outcome in the OAC + AP group was comparable to that in the OAC alone group (HR, 1.28 [0.93–1.78]; p = 0.13), whereas among those in the low or intermediate score subgroups, the risk of primary outcome in the OAC + AP group was significantly increased compared with that in the OAC alone group (Table 4).

**Table 4:** Association of OAC vs OAC + AP with primary outcome according to the CHA2DS2-VASc score subgroup.

	Crude HR	*p*	Pint	Cox PH*	*p*	Pint	IPTW^a^	*p*	Pint
**All anticoagulated**									
OAC	Ref			Ref			Ref		
OAC + AP	1.61 (1.34–1.93)	< 0.001	0.04	1.48 (1.22–1.78)	< 0.001	0.03	1.70 (1.35–2.13)	< 0.001	0.01
**Low score**									
OAC	Ref			Ref			Ref		
OAC + AP	2.41 (1.51–3.82)	0.0002		2.56 (1.61–4.08)	< 0.001		3.51 (1.94–6.36)	< 0.001	
**Intermediate score**									
OAC	Ref			Ref			Ref		
OAC + AP	1.48 (1.11–1.97)	0.01		1.53 (1.14–2.05)	0.004		1.57 (1.13–2.19)	0.01	
**High score**									
OAC	1(ref)			1(ref)			1(ref)		
OAC + AP	1.19 (0.91–1.57)	0.21		1.24 (0.94–1.64)	0.13		1.28 (0.93–1.75)	0.13	

*Adjusted variables: age, NIHSS scores, dyslipidemia, smoking, prior anticoagulant use, prior statin use, prior antihypertensive use, prior antidiabetic use, LAD, glucose, SBP, LDL-cholesterol, multiple lesions, reperfusion therapy, stroke mechanism, CHA2DS2-VASc score.

^a^Weighted Cox proportional hazards model with robust standard errors.

Kaplan–Meier cumulative incidence plots of the primary outcome are shown in Fig. 2. The outcome difference between the OAC + AP and OAC groups was consistent for 1 year after stroke in all subjects and in all CHA2DS2-VASc score subgroups, though an interaction existed between subgroups and OAC at discharge.

## Discussion

In this nationwide, multicenter registry-based analysis of over 7000 patients with AIS and AF, there were significant associations of increasing CHA2DS2-VASc scores with increasing risk of primary outcome events, recurrent stroke, and all-cause mortality at 1 year, especially in OAC-treated patients. In addition, among anticoagulated patients after AIS, though a potential interaction between CHA2DS2-VASc score subgroups and OAC treatment (OAC/OAC + AP) was observed, there was lack of evidence supporting which OAC + AP treatment could reduce the risk of vascular events in the high CHA2DS2-VASc score subgroup. The findings, however, should be interpreted with consideration of the key limitations of our study methodology.

Our results provide important information on the estimated risk of vascular events according to the CHA2DS2-VASc score in real-world cohorts of AIS patients with AF considering OAC treatment. Compared with the results of the Danish registry, in which patients were discharged with AF and no anticoagulation therapy^12^, estimated event rates of recurrent stroke and all-cause mortality at 1 year were substantially low in our study populations, especially in anticoagulated and high-scoring patients. However, our results were comparable to the results of the larger Swedish registry with 90,490 AF patients without warfarin throughout follow-up^13^. Although not being able to directly compare with a previous study, among patients with the lowest risk of score 0 prior to stroke, the estimated risks were similar to those of previous non-anticoagulated AF cohorts (0.8% for thromboembolism and 4.9% for all-cause mortality in the Danish cohort)^12^. However, in our study, the results indicating that non-anticoagulated AIS patients had substantially higher estimated event rates than anticoagulated patients should be interpreted with caution because of selection bias.

Nonetheless, we found that among patients treated with OAC at discharge, a risk stratification scheme using the CHA2DS2-VASc score might be possible. In the unadjusted analysis, with every 1-point increase in the CHA2DS2-VASc score, relative increased risks of 31% for primary outcome events, 25% for recurrent stroke, and 36% for all-cause mortality were estimated. These results seemed to imply the potential usefulness of the CHA2DS2-VASc score for vascular event risk stratification in anticoagulated patients with AF after AIS. Our results were concordant with previous studies in which, in AIS with or without AF, the CHA2DS2-VASc scores could be helpful for predicting clinical outcomes such as 2-year death/disability, 2-year mortality, and ischemic or hemorrhagic events within 2 years^14^. Given that the stroke with AF population would have more risk factors than the AF only population, it is noteworthy to show how high risks could be estimated in AIS populations with anticoagulation. However, our study differed from the previous study in investigating the estimated risks of vascular events according to the individual CHA2DS2-VASc scores in AIS and AF using multicenter registry. In previous studies of stroke populations, there were limitations to assessing only 3-month short-term events or functional outcomes after stroke or investigating small samples^15,16^.

On the other hand, our results suggest that OAC-only treatment might not be sufficient for the prevention of vascular events in the high CHA2DS2-VASc score group. Our study found that as the CHA2DS2-VASc score increased, the proportion of OAC + AP treatment also increased, up to over 30% of patients with CHA2DS2-VASc scores of 7 or more. It is likely that physicians weigh the perceived atherothrombotic burden when deciding on antithrombotic treatment. These results provide important information about real-world practice from a multicenter registry and will be helpful in planning future trials.

As it is well-known that high CHADS2 scores are associated with high atherosclerotic burdens^7^, the combination of OAC and AP might have been considered to reduce the risk of both embolic events and thrombotic events. In our study, the magnitude of the relative risk for primary outcome events in the OAC + AP group vs OAC alone was dependent upon the CHA2DS2-VASc score subgroups, meaning there was a quantitative interaction between the CHA2DS2-VASc score subgroups and OAC treatment types. These results, however, may be only weak evidence supporting the addition of AP to OAC considering the CHA2DS2-VASc score, as OAC + AP treatment, even in the high CHA2DS2-VASc score subgroup, was associated with a numerically higher risk of primary outcome events (weighted HR 1.28 [0.93–1.75]).

However, the main limitations for studying the effectiveness of OAC + AP treatment in contemporary cohorts of AIS and AF patients are the major selection bias for which it can be only partly adjusted, since there will be measured and unmeasured confounders related to why these patients were taking OAC + AP in the first place. Nonetheless, in a recent study, an analysis restricted to patients with AF and ipsilateral stroke with significant large artery stenosis, not complete occlusion, showed that OAC + AP was less likely to be associated with 3-month vascular events than OAC alone (weighted HR 0.25 [0.07–0.89])^17^. Therefore, the results should be interpreted with caution and limited to a hypothesis generation function. These findings, however, suggest that future trials for secondary prevention in patients with AIS with AF might need to focus on patients with a high CHA2DS2-VASc score subgroup.

In addition, we presented the estimated risks according to two different CHA2DS2-VASc scores considering index stroke and history of stroke or TIA. Given that 2 points should be added to the CHA2DS2-VASc scores after stroke if there is no history of stroke or TIA, showing the estimated event rates according to poststroke CHA2DS2-VASc scores would be noteworthy. As expected, the poststroke CHA2DS2-VASc score of 2, the lowest score in this scoring system, was slightly higher than the CHA2DS2-VASc score of 0 (primary outcome; 6.9% vs 6.0%, respectively). As the original CHA2DS2-VASc score estimated the risk of thromboembolism or death in non-anticoagulated patients with AF, the relatively lower expected vascular event risk of poststroke CHA2DS2-VASc scores in our cohorts might be partly explained by anticoagulated patients with AIS and AF.

There were several limitations to this study. First, it was a registry-based study with treatment selection based on clinician decision-making rather than random allocation. Although PS analysis was used to mitigate baseline imbalances between treatment groups, the possibility of residual confounding remains. Second, the patient cohort was restricted to a South Korean population, though it was a multicenter, nationwide study; studies in other racial/ethnic groups are needed to confirm the generalizability of our conclusions. Third, structured follow-up interviews were not designed to reliably identify all possible adverse bleeding events associated with antithrombotic therapy, though more general and important safety outcomes of all-cause mortality were considered. Fourth, compliance with and duration of antithrombotic therapy regimens were not ascertained by direct interviews. More importantly, our lack of data on anticoagulation control (e.g., time in therapeutic range, TTR) or dose/intensity of OAC or AP may well be a limitation. Nevertheless, our study was noteworthy and thoroughly explored the clinical implications of the CHA2DS2-VASc score in patients with AIS and AF from a prospective multicenter stroke registry. Our results therefore expand the understanding of the CHA2DS2-VASc score in patients with AIS and AF.

## Conclusion

In analyses of real-world, multicenter registry-based AIS patients with AF, our study found that the risk of vascular events increased with increasing CHA2DS2-VASc score in OAC-treated patients with AIS and AF; in addition, our results suggest that OAC alone treatment could still be preferred over OAC + AP treatment for secondary prevention in AIS patients with AF, though a potential interaction between the CHA2DS2-VASc score subgroups and OAC at discharge (OAC and OAC + AP) was observed on primary outcome events. However, our study addresses a need for future randomized studies to tailor optimal antithrombotic treatment in patients with AIS and AF with high atherothrombotic risks.

## Supplementary Information

Supplementary Information.

## Data Availability

Data used in this study are available upon reasonable request following submission of a legitimate academic research proposal to be assessed by the CRCS-K steering committee.
