# Supplementary material for: CHA2DS2-VASc score in acute ischemic stroke with atrial fibrillation: results from the Clinical Research Collaboration for Stroke in Korea
Source: Sci Rep. 2021 Jan 12;11:793. doi: 10.1038/s41598-020-80874-1 (PMC7804950; doi:10.1038/s41598-020-80874-1)
Supplement: Supplementary file 1 — Supplementary Information. [file 41598_2020_80874_MOESM1_ESM.docx]

**ONLINE SUPPLEMENTS**

Title: CHA2DS2-VASc score in acute ischemic stroke with atrial fibrillation: results from the Clinical Research Collaboration for Stroke in Korea

Hak-Loh Lee, MD,^a^ Joon-Tae Kim, MD, PhD,^a^ Ji Sung Lee, PhD,^b^ Beom Joon Kim, MD, PhD,^c^ Jong-Moo Park, MD, PhD,^d^ Kyusik Kang, MD, PhD,^d^ Soo Joo Lee, MD, PhD,^e^ Jae Guk Kim, MD, ^e^ Jae-Kwan Cha, MD, PhD,^f^ Dae-Hyun Kim, MD, PhD,^f^ Tai Hwan Park, MD, PhD,^g^ Sang-Soon Park, MD,^g^ Kyung Bok Lee, MD, PhD,^h^ Jun Lee, MD, PhD,^i^ Keun-Sik Hong, MD, PhD,^j^ Yong-Jin Cho, MD, PhD,^j^ Hong-Kyun Park, MD,^j^ Byung-Chul Lee, MD, PhD,^k^ Kyung-Ho Yu, MD, PhD,^k^ Mi Sun Oh, MD, PhD, ^k^ Dong-Eog Kim, MD, PhD,^l^ Wi-Sun Ryu, MD,^l^ Jay Chol Choi, MD, PhD,^m^ Jee-Hyun Kwon, MD, PhD,^n^ Wook-Joo Kim, MD, PhD,^n^ Dong-Ick Shin, MD, PhD,^o^ Sung Il Sohn, MD, PhD,^p^ Jeong-Ho Hong, MD, PhD,^p^ Man-Seok Park, MD, PhD,^a^ Kang-Ho Choi, MD, PhD,^a^ Ki-Hyun Cho, MD, PhD,^a^ Juneyoung Lee, PhD,^q^ Hee-Joon Bae, MD, PhD.^c^

^a^Department of Neurology, Chonnam National University Hospital, Chonnam National University Medical School, Gwangju, Korea

^b^Clinical Research Center, Asan Institute for Life Sciences, Asan Medical Center, University of Ulsan College of Medicine, Seoul, Korea

^c^Department of Neurology, Cerebrovascular Center, Seoul National University Bundang Hospital, Seongnam, Korea

^d^Department of Neurology, Nowon Eulji Medical Center, Eulji University, Seoul, Korea

^e^Department of Neurology, Eulji University Hospital, Eulji University, Daejeon, Korea

^f^Department of Neurology, Dong-A University Hospital, Busan, Korea

^g^Department of Neurology, Seoul Medical Center, Seoul, Korea

^h^Department of Neurology, Soonchunhyang University Hospital, Seoul, Korea

^i^Department of Neurology, Yeungnam University Hospital, Daegu, Korea

^j^Department of Neurology, Ilsan Paik Hospital, Inje University, Goyang, Korea

^k^Department of Neurology, Hallym University Sacred Heart Hospital, Anyang, Korea

^l^Department of Neurology, Dongguk University Ilsan Hospital, Goyang, Korea

^m^Department of Neurology, Jeju National University Hospital, Jeju National University School of Medicine, Jeju, Korea

^n^Department of Neurology, Ulsan University College of Medicine, Ulsan, Korea

^o^Department of Neurology, Chungbuk National University Hospital, Cheongju, Korea

^p^Department of Neurology, Keimyung University Dongsan Medical Center, Daegu, Korea

^q^Department of Biostatistics, Korea University College of Medicine, Seoul, Korea

Supplemental Methods

Supplemental Figure I

Supplemental Tables I-X

Corresponding author: Joon-Tae Kim

Department of Neurology, Chonnam National University Medical School,

Gwangju-Jeonnam Regional Cerebrovascular Center, Chonnam National University Hospital

42 Jebongro, Dong-gu, Gwangju, 61469, Korea

Phone: 82-62-220-6180

Fax: 82-62-228-3461

E-mail: [alldelight2@jnu.ac.kr](mailto:alldelight2@jnu.ac.kr)

Cocorresponding author: Hee-Joon Bae

Department of Neurology, Seoul National University College of Medicine

Cerebrovascular Center, Seoul National University Bundang Hospital

82, Gumi-ro 173 Beon-gil, Bundang-gu, Seongnam-si, Gyeonggi-do, 13620, Korea

Phone: 82-31-787-7467

Fax: 82-31-787-4059

E-mail: [braindoc@snu.ac.kr](mailto:braindoc@snu.ac.kr)

Supplemental Methods

(A) Data collection

Demographic, clinical, imaging, and laboratory data were prospectively collected as previously reported. Baseline data, including National Institutes of Health Stroke Scale (NIHSS) scores, were collected from all patients. The following data were directly obtained from the registry database: (1) demographic information, including age, sex, and body mass index (BMI); (2) medical history, including previous transient ischemic attack (TIA), previous stroke, previous coronary artery disease (CAD), previous peripheral artery disease (PAD), hypertension, diabetes mellitus, dyslipidemia, smoking (recent), and congestive heart failure; (3) medication, including previous antiplatelet medication, previous anticoagulant medication, previous antihypertensive medication, previous antidiabetic treatment, and previous statin medication; (4) stroke characteristics and acute treatment, including the arrival time, initial NIHSS scores, reperfusion therapy, multiple lesions, large arterial steno-occlusion (LAD), which was categorized as no stenosis, mild stenosis (<50%), moderate-to-severe stenosis (≥50%) and complete occlusion, and the CHA2DS2-VASc score; (5) laboratory data, including white blood cell (WBC) counts, hemoglobin, platelet counts, creatinine serum levels, glucose at presentation, fasting low-density lipoprotein (LDL), prothrombin time (international normalized ratio, INR), and systolic blood pressure; and (6) in-hospital treatment, including antihypertensive therapies, statin therapies, and antidiabetic therapies. If less than 5% of the continuous values were missing, the data were imputed as median values. The CHA2DS2-VASc score was the sum of points after the addition of one point each for heart failure, hypertension, diabetes, vascular disease, age 65-74 years, and female sex and two points each for previous stroke or TIA and age ≥75 years. We considered two different CHA2DS2-VASc scores: the CHA2DS2-VASc score, which did not add 2 points for the index stroke, and the poststroke CHA2DS2-VASc score, which considered 2 points for the index stroke if there was no previous stroke or TIA. This score thus ranged from 0 to 9 for the CHA2DS2-VASc score and 2 to 9 for the poststroke CHA2DS2-VASc score. The CHA2DS2-VASc score was categorized further as low (0-2), intermediate (3-4), and high (5 or more) scores considering the similar proportions of the population. Antithrombotic regimens at discharge were categorized into no OAC (no antithrombotics or AP only) and OAC with or without AP (OAC alone and OAC+AP).

(B) Outcomes

Vascular events were prospectively observed during a 3-month follow-up period after the qualifying event via routine clinic visits or telephone interviews performed by dedicated nurses or physicians with a predefined protocol. To ensure the accuracy of the outcome and to minimize interinterviewer discrepancy, a set of uniform, structured questionnaires were administered by trained personnel.

(C) Statistical analysis

The frequency (percentage), mean (± standard deviation, SD), or median (interquartile range, IQR) are reported depending on the variable type. Categorical variables were analyzed using Pearson’s chi-square test or Fisher’s exact test, and continuous variables were analyzed using Student’s t-test or the Wilcoxon rank-sum test, as appropriate.

We estimated event rates for primary vascular event outcome, recurrent stroke, and all-cause mortality for the individual CHA2DS2-VASc scores and poststroke CHA2DS2-VASc score by Kaplan-Meier estimates. We also investigated the risk of primary vascular event outcome by using Cox proportional hazard regression models. Adjustment variables were predetermined based on the clinical relevance as follows: age, initial NIHSS scores, and prior anticoagulation.

For the analysis of the effectiveness of OAC vs OAC+AP, we used the cohort propensity score (PS) in the subgroup analyses method to generate PS. This method adds an interaction term between the CHA2DS2-VASc score subgroup variable or the type of OAC at discharge, which is an effect modifier, and other covariates to the PS model. This score was used in the subsequent multivariable analyses for an outcome model with the inverse probability of treatment weighting (IPTW) method; in this method, the subjects in the OAC+AP group were weighted by the inverse of their PSs, whereas those in the OAC group were weighted by an inverse of 1 - PS. An absolute standardized difference <0.1 for a baseline covariate indicated a relatively low imbalance between the groups.

The event rates of primary and secondary outcome measures were estimated using the weighted Kaplan-Meier method and compared between the OAC and OAC+AP groups using the IPTW log-rank. Weighted Cox proportional hazards regression with robust standard error was used to estimate and compare the time to event between the OAC and OAC+AP groups. Supplemental analyses were performed with Cox proportional hazard models using adjustment for variables that were predetermined based on their influence on the outcomes and treatment groups (i.e., age, NIHSS scores, dyslipidemia, smoking, prior anticoagulant use, prior statin use, prior antihypertensive use, prior antidiabetic use, LAD, glucose, SBP, LDL cholesterol, multiple lesion, reperfusion therapy, stroke mechanism, and CHA2DS2-VASc score). Adjusted incidence rate differences were estimated using weighted event counts and follow-up times within cohorts. Statistical significance was determined using 95% confidence intervals (CIs) and 2-tailed P-values (P ≤0.05). For interaction testing, considering the known insensitivity of interaction testing, P-values <0.10 indicated heterogeneity. Analyses were performed using SAS v. 9.4 (SAS Institute Inc., Cary, NC).

Supplemental Figure 1. Selection of the study population

59,512 patients with acute ischemic stroke in the CRCS-K registry (Jan. 2011 - Nov. 2017)

52,117 excluded

Admission not within 48 h of onset (n=11,904)

No ischemic lesions on the ADC (n=3,642)

No AF (n=33,820)

Initial NIHSS >15 (n=2,751)

7,395 acute, mild-to-moderate ischemic stroke with AF within 48 h of onset (main analysis)

1,895 excluded

No OAC therapy at discharge (n=1,895)

5,500 OAC-treated patients included in the secondary analysis

Supplemental Table I. Cumulative vascular event rates at 1 year (%) according to CHA2DS2-VASc scores

|  | All patients (N=7395) | | Nonanticoagulated patients (n=1895) | | Anticoagulated patients (n=5500) | |
| --- | --- | --- | --- | --- | --- | --- |
| Recurrent stroke |  |  |  |  |  |  |
| 0 | 4 | 1.43 (0.04-2.82) | 2 | 2.90 (0.00-6.86) | 2 | 0.95 (0.00-2.25) |
| 1 | 21 | 3.11 (1.79-4.42) | 10 | 7.08 (2.82-11.34) | 11 | 2.08 (0.86-3.30) |
| 2 | 49 | 4.38 (3.14-5.62) | 22 | 9.00 (5.30-12.70) | 27 | 3.08 (1.89-4.27) |
| 3 | 79 | 5.46 (4.27-6.64) | 35 | 10.21 (6.93-13.49) | 44 | 3.95 (2.79-5.11) |
| 4 | 85 | 5.70 (4.48-6.91) | 34 | 9.77 (6.55-12.98) | 51 | 4.39 (3.16-5.63) |
| 5 | 76 | 7.34 (5.69-8.99) | 25 | 8.59 (5.18-11.99) | 51 | 6.75 (4.91-8.58) |
| 6 | 48 | 8.86 (6.37-11.34) | 19 | 13.66 (7.77-19.56) | 29 | 7.11 (4.50-9.73) |
| 7 or more | 25 | 10.03 (6.16-13.91) | 12 | 16.97 (7.39-26.54) | 13 | 7.51 (3.54-11.47) |
| All-cause mortality |  |  |  |  |  |  |
| 0 | 14 | 4.63 (2.15-7.11) | 11 | 16.36 (7.51-25.21) | 3 | 1.12 (0.00-2.69) |
| 1 | 34 | 4.78 (3.18-6.39) | 27 | 17.64 (11.44-23.84) | 7 | 1.39 (0.35-2.43) |
| 2 | 107 | 10.04 (8.20-11.87) | 66 | 28.01 (22.15-33.87) | 41 | 5.05 (3.52-6.57) |
| 3 | 215 | 14.67 (12.82-16.51) | 140 | 36.61 (31.66-41.56) | 75 | 7.04 (5.48-8.60) |
| 4 | 265 | 17.16 (15.23-19.08) | 154 | 37.48 (32.68-42.27) | 111 | 9.87 (8.08-11.67) |
| 5 | 188 | 17.68 (15.33-20.02) | 103 | 36.08 (30.39-41.76) | 85 | 10.96 (8.70-13.22) |
| 6 | 122 | 22.13 (18.56-25.70) | 64 | 41.43 (33.49-49.38) | 58 | 14.85 (11.20-18.51) |
| 7 or more | 70 | 26.29 (20.89-31.68) | 37 | 45.71 (34.45-56.96) | 33 | 18.16 (12.50-23.81) |

All P-values <0.001 by log-rank test

Supplemental Table II. Adjusted cumulative event rates at 1 year according to CHA2DS2-VASc scores

|  | All patients (N=7395) | |  | Nonanticoagulated patients (n=1895) | |  | Anticoagulated patients (n=5500) | |  |
| --- | --- | --- | --- | --- | --- | --- | --- | --- | --- |
| CHA2DS2-VASc score | No. events | Event Rate  (%, 95% CI)^a^ | P-value | No. events | Event Rate  (%, 95% CI)^a^ | P-value | No. events | Event Rate  (%, 95% CI)^a^ | P-value |
| (A) Primary outcome |  |  | 0.003 |  |  | 0.77 |  |  | <0.001 |
| 0 | 18 | 10.07 (5.39-14.52) |  | 13 | 34.11 (16.52-48.00) |  | 5 | 3.96 (0.42-7.37) |  |
| 1 | 53 | 9.80 (7.13-12.40) |  | 37 | 35.01 (24.61-43.98) |  | 16 | 4.17 (2.09-6.21) |  |
| 2 | 151 | 13.08 (11.04-15.06) |  | 84 | 36.93 (30.26-42.97) |  | 67 | 8.40 (6.43-10.33) |  |
| 3 | 270 | 14.09 (12.41-15.74) |  | 159 | 37.88 (32.97-42.43) |  | 111 | 8.88 (7.23-10.50) |  |
| 4 | 316 | 13.67 (12.04-15.27) |  | 174 | 33.49 (28.91-37.78) |  | 142 | 9.69 (7.99-11.36) |  |
| 5 | 250 | 16.05 (14.00-18.05) |  | 124 | 36.41 (30.86-41.51) |  | 126 | 12.27 (10.02-14.46) |  |
| 6 | 152 | 17.87 (15.01-20.63) |  | 72 | 38.25 (30.60-45.06) |  | 80 | 14.13 (10.91-17.23) |  |
| 7 or more | 84 | 19.32 (15.27-23.16) |  | 43 | 41.75 (31.16-50.71) |  | 41 | 15.00 (10.36-19.41) |  |
| (B) Recurrent stroke |  |  | <0.001 |  |  | 0.19 |  |  | <0.001 |
| 0 | 4 | 1.17 (0.00-2.33) |  | 2 | 2.73 (0.00-6.52) |  | 2 | 0.66 (0.00-1.60) |  |
| 1 | 21 | 2.59 (1.40-3.77) |  | 10 | 5.93 (1.89-9.80) |  | 11 | 1.55 (0.57-2.53) |  |
| 2 | 49 | 4.42 (3.15-5.66) |  | 22 | 9.36 (5.39-13.17) |  | 27 | 2.94 (1.80-4.07) |  |
| 3 | 79 | 5.54 (4.34-6.73) |  | 35 | 9.62 (6.49-12.64) |  | 44 | 4.04 (2.85-5.21) |  |
| 4 | 85 | 5.91 (4.64-7.15) |  | 34 | 8.77 (5.80-11.65) |  | 51 | 4.80 (3.47-6.10) |  |
| 5 | 76 | 7.76 (6.00-9.48) |  | 25 | 8.95 (5.43-12.33) |  | 51 | 7.08 (5.10-9.02) |  |
| 6 | 48 | 9.57 (6.84-12.22) |  | 19 | 13.31 (7.31-18.93) |  | 29 | 7.84 (4.92-10.67) |  |
| 7 or more | 25 | 10.87 (6.60-14.95) |  | 12 | 16.24 (7.10-24.48) |  | 13 | 8.18 (3.66-12.48) |  |
| (C) All-cause mortality |  |  | 0.03 |  |  | 0.70 |  |  | 0.02 |
| 0 | 14 | 9.12 (4.28-13.72) |  | 11 | 32.09 (13.82-46.49) |  | 3 | 3.69 (0.00-7.78) |  |
| 1 | 34 | 6.75 (4.43-9.01) |  | 27 | 28.20 (18.03-37.11) |  | 7 | 2.40 (0.60-4.16) |  |
| 2 | 107 | 8.56 (6.93-10.17) |  | 66 | 29.51 (23.14-35.35) |  | 41 | 5.03 (3.49-6.55) |  |
| 3 | 215 | 9.49 (8.13-10.83) |  | 140 | 32.42 (27.65-36.88) |  | 75 | 5.02 (3.81-6.21) |  |
| 4 | 265 | 9.15 (7.85-10.43) |  | 154 | 28.07 (23.71-32.18) |  | 111 | 5.72 (4.46-6.96) |  |
| 5 | 188 | 9.99 (8.43-11.52) |  | 103 | 30.19 (24.87-35.14) |  | 85 | 6.35 (4.83-7.85) |  |
| 6 | 122 | 11.61 (9.41-13.75) |  | 64 | 33.14 (25.79-39.76) |  | 58 | 7.60 (5.42-9.73) |  |
| 7 or more | 70 | 12.79 (9.71-15.77) |  | 37 | 35.73 (25.50-44.55) |  | 33 | 8.57 (5.44-11.60) |  |

^a^ based on the Cox PH regression

Adjusted variables: age, initial NIHSS score, prior anticoagulation

P-value by Cox PH regression

Supplemental Table III. Cumulative event rates at 1 year according to poststroke CHA2DS2-VASc scores

|  | All patients (N=7395) | |  | Nonanticoagulated patients (n=1895) | |  | Anticoagulated patients (n=5500) | |  |
| --- | --- | --- | --- | --- | --- | --- | --- | --- | --- |
| Poststroke CHA2DS2-VASc score | No. events | Event Rate  (%, 95% CI)a | P-value | No. events | Event Rate  (%, 95% CI)a | P-value | No. events | Event Rate  (%, 95% CI)a | P-value |
| (A) Primary outcome |  |  | <0.001 |  |  | <0.001 |  |  | <0.001 |
| 2 | 24 | 6.90 (4.16-9.64) |  | 16 | 20.67 (11.59-29.76) |  | 8 | 2.84 (0.74-4.95) |  |
| 3 | 78 | 9.07 (7.11-11.02) |  | 49 | 25.70 (19.38-32.02) |  | 29 | 4.41 (2.80-6.02) |  |
| 4 | 187 | 13.79 (11.92-15.67) |  | 95 | 31.69 (26.34-37.04) |  | 92 | 8.71 (6.96-10.47) |  |
| 5 | 368 | 18.93 (17.15-20.70) |  | 204 | 39.83 (35.50-44.16) |  | 164 | 11.55 (9.85-13.24) |  |
| 6 | 396 | 22.39 (20.39-24.38) |  | 215 | 43.46 (39.01-47.92) |  | 181 | 14.24 (12.25-16.23) |  |
| 7 | 194 | 26.05 (22.84-29.25) |  | 97 | 44.05 (37.37-50.74) |  | 97 | 18.64 (15.24-22.03) |  |
| 8 or more | 47 | 34.72 (26.56-42.88) |  | 30 | 64.55 (50.42-78.67) |  | 17 | 19.63 (11.16-28.10) |  |
| (B) Recurrent stroke |  |  | 0.002 |  |  | 0.11 |  |  | 0.02 |
| 2 | 7 | 2.08 (0.55-3.61) |  | 4 | 5.26 (0.22-10.30) |  | 3 | 1.16 (0.00-2.48) |  |
| 3 | 32 | 3.94 (2.59-5.28) |  | 12 | 6.95 (3.13-10.77) |  | 20 | 3.13 (1.77-4.48) |  |
| 4 | 65 | 4.77 (3.59-5.96) |  | 23 | 8.04 (4.77-11.30) |  | 42 | 3.87 (2.65-5.08) |  |
| 5 | 113 | 6.04 (4.93-7.14) |  | 45 | 9.63 (6.83-12.42) |  | 68 | 4.84 (3.70-5.99) |  |
| 6 | 107 | 6.44 (5.22-7.65) |  | 47 | 11.36 (8.19-14.52) |  | 60 | 4.74 (3.52-5.96) |  |
| 7 | 50 | 7.28 (5.27-9.28) |  | 20 | 10.48 (5.89-15.07) |  | 30 | 6.05 (3.90-8.20) |  |
| 8 or more | 13 | 10.36 (4.85-15.88) |  | 8 | 21.68 (7.70-35.66) |  | 5 | 5.79 (0.71-10.88) |  |
| (C) All-cause mortality |  |  | <0.001 |  |  | <0.001 |  |  | <0.001 |
| 2 | 18 | 4.90 (2.54-7.27) |  | 13 | 16.03 (7.65-24.41) |  | 5 | 1.70 (0.02-3.38) |  |
| 3 | 51 | 5.93 (4.31-7.55) |  | 39 | 21.10 (15.07-27.12) |  | 12 | 1.80 (0.73-2.86) |  |
| 4 | 130 | 10.05 (8.40-11.71) |  | 76 | 26.86 (21.63-32.08) |  | 54 | 5.43 (4.01-6.85) |  |
| 5 | 282 | 14.91 (13.27-16.55) |  | 174 | 35.43 (31.10-39.76) |  | 108 | 7.80 (6.35-9.24) |  |
| 6 | 335 | 19.46 (17.54-21.39) |  | 190 | 39.81 (35.30-44.32) |  | 145 | 11.77 (9.91-13.64) |  |
| 7 | 160 | 22.25 (19.16-25.35) |  | 82 | 39.22 (32.43-46.01) |  | 78 | 15.50 (12.30-18.70) |  |
| 8 or more | 39 | 30.02 (22.01-38.04) |  | 28 | 62.51 (47.86-77.16) |  | 11 | 13.55 (6.05-21.05) |  |

Supplemental Table IV. Association of CHA2DS2-VASc scores with secondary vascular outcomes in the OAC treatment group (n=5500)

|  | Crude HR (95% CI) | P | Model 1 (HR, 95% CI) | P | Model 2 (HR, 95% CI) | P |
| --- | --- | --- | --- | --- | --- | --- |
| Recurrent stroke |  |  |  |  |  |  |
| Per 1-point increase | 1.25 (1.16-1.35) | <0.001 | 1.29 (1.18-1.40) | <0.001 | 1.26 (1.15-1.39) | <0.001 |
| 0 | ref |  | ref |  | ref |  |
| 1 | 2.18 (0.48-9.85) | 0.31 | 2.30 (0.51-10.37) | 0.28 | 2.26 (0.50-10.23) | 0.29 |
| 2 | 3.60 (0.86-15.15) | 0.08 | 4.17 (0.98-17.67) | 0.05 | 4.04 (0.95-17.20) | 0.06 |
| 3 | 4.50 (1.09-18.57) | 0.04 | 5.54 (1.32-23.25) | 0.02 | 5.30 (1.25-22.42) | 0.02 |
| 4 | 5.05 (1.23-20.75) | 0.02 | 6.44 (1.53-27.13) | 0.01 | 6.13 (1.44-26.18) | 0.01 |
| 5 | 7.50 (1.83-30.81) | 0.01 | 9.62 (2.28-40.68) | 0.002 | 8.86 (2.06-38.12) | 0.003 |
| 6 | 8.21 (1.96-34.42) | 0.004 | 10.60 (2.45-45.93) | 0.002 | 9.83 (2.23-43.38) | 0.003 |
| 7 or more | 8.16 (1.84-36.15) | 0.01 | 10.92 (2.38-50.21) | 0.002 | 9.89 (2.09-46.66) | 0.004 |
| All-cause mortality |  |  |  |  |  |  |
| Per 1 point increase | 1.36 (1.28-1.44) | <0.001 | 1.19 (1.11-1.27) | <0.001 | 1.24 (1.16-1.32) | <0.001 |
| 0 | ref |  | ref |  | ref |  |
| 1 | 0.93 (0.24-3.62) | 0.92 | 0.71 (0.18-2.76) | 0.62 | 0.83 (0.21-3.23) | 0.79 |
| 2 | 3.69 (1.14-11.91) | 0.03 | 1.81 (0.56-5.87) | 0.33 | 2.23 (0.68-7.25) | 0.18 |
| 3 | 5.19 (1.64-16.47) | 0.01 | 1.97 (0.61-6.31) | 0.26 | 2.53 (0.78-8.15) | 0.12 |
| 4 | 7.40 (2.35-23.29) | 0.001 | 2.39 (0.75-7.65) | 0.14 | 3.32 (1.03-10.67) | 0.04 |
| 5 | 8.45 (2.67-26.72) | 0.0003 | 2.66 (0.83-8.54) | 0.10 | 3.92 (1.21-12.69) | 0.02 |
| 6 | 11.12 (3.48-35.48) | <0.001 | 3.28 (1.01-10.65) | 0.048 | 4.56 (1.39-14.94) | 0.01 |
| 7 or more | 14.09 (4.32-45.95) | <0.001 | 3.66 (1.10-12.17) | 0.03 | 5.76 (1.71-19.41) | 0.005 |

Model 1: age, initial NIHSS, prior anticoagulation

Mode 2: age, initial NIHSS, BMI, arrival time, dyslipidemia, smoking, prior anticoagulation, prior statin, antihypertensive treatment, antidiabetic treatment, statin treatment, reperfusion therapy, creatinine, glucose, SBP, large artery diseases, multiple lesions, stroke mechanism (TOAST)

Supplemental Table V. Association of CHA2DS2-VASc scores with vascular events in all patients

|  | Crude HR (95% CI) | P | Model 1 (HR, 95% CI) | P | Model 2 (HR, 95% CI) | P |
| --- | --- | --- | --- | --- | --- | --- |
| (A) Primary outcome |  |  |  |  |  |  |
| Per 1-point increase | 1.23 (1.19-1.27) | <0.001 | 1.10 (1.06-1.15) | <0.001 | 1.12 (1.07-1.17) | <0.001 |
| 0 | Ref | 13 | ref |  | ref |  |
| 1 | 1.19 (0.70-2.03) | 0.53 | 0.97 (0.57-1.66) | 0.92 | 1.07 (0.63-1.84) | 0.79 |
| 2 | 2.29 (1.41-3.73) | 0.001 | 1.32 (0.80-2.18) | 0.28 | 1.52 (0.92-2.52) | 0.10 |
| 3 | 3.04 (1.89-4.89) | <0.001 | 1.43 (0.87-2.35) | 0.16 | 1.71 (1.03-2.83) | 0.04 |
| 4 | 3.38 (2.10-5.43) | <0.001 | 1.39 (0.84-2.29) | 0.20 | 1.74 (1.04-2.92) | 0.03 |
| 5 | 3.96 (2.46-6.39) | <0.001 | 1.65 (0.99-2.73) | 0.05 | 2.05 (1.22-3.45) | 0.01 |
| 6 | 4.68 (2.87-7.62) | <0.001 | 1.85 (1.10-3.12) | 0.03 | 2.29 (1.34-3.92) | 0.002 |
| 7 or more | 5.56 (3.34-9.25) | <0.001 | 2.02 (1.18-3.47) | 0.01 | 2.49 (1.42-4.36) | 0.002 |
| (B) Recurrent stroke |  |  |  |  |  |  |
| Per 1-point increase | 1.22 (1.15-1.29) | <0.001 | 1.25 (1.16-1.34) | <0.001 | 1.23 (1.13-1.33) | <0.001 |
| 0 | ref |  | ref |  | ref |  |
| 1 | 2.13 (0.73-6.19) | 0.17 | 2.23 (0.77-6.51) | 0.14 | 2.19 (0.75-6.39) | 0.15 |
| 2 | 3.33 (1.20-9.24) | 0.02 | 3.84 (1.37-10.78) | 0.01 | 3.77 (1.34-10.64) | 0.01 |
| 3 | 3.97 (1.46-10.85) | 0.01 | 4.85 (1.73-13.59) | 0.003 | 4.68 (1.65-13.26) | 0.004 |
| 4 | 4.08 (1.50-11.12) | 0.01 | 5.18 (1.83-14.65) | 0.002 | 4.98 (1.73-14.33) | 0.003 |
| 5 | 5.37 (1.97-14.69) | 0.001 | 6.86 (2.41-19.53) | 0.0003 | 6.41 (2.20-18.67) | 0.001 |
| 6 | 6.58 (2.37-18.24) | 0.0003 | 8.55 (2.94-24.86) | <0.001 | 7.89 (2.65-23.49) | 0.0002 |
| 7 or more | 7.33 (2.55-21.05) | 0.0002 | 9.78 (3.24-29.59) | <0.001 | 8.61 (2.75-26.95) | 0.0002 |
| (C) All-cause mortality |  |  |  |  |  |  |
| Per 1-point increase | 1.25 (1.21-1.30) | <0.001 | 1.08 (1.04-1.13) | 0.001 | 1.12 (1.07-1.18) | <0.001 |
| 0 | ref |  | ref |  | ref |  |
| 1 | 0.98 (0.53-1.83) | 0.96 | 0.73 (0.39-1.36) | 0.32 | 0.84 (0.45-1.58) | 0.59 |
| 2 | 2.10 (1.20-3.66) | 0.01 | 0.94 (0.53-1.66) | 0.82 | 1.12 (0.63-1.99) | 0.70 |
| 3 | 3.12 (1.82-5.36) | <0.001 | 1.04 (0.59-1.84) | 0.89 | 1.33 (0.75-2.36) | 0.33 |
| 4 | 3.65 (2.13-6.24) | <0.001 | 1.00 (0.57-1.78) | 0.99 | 1.39 (0.77-2.49) | 0.27 |
| 5 | 3.85 (2.24-6.63) | <0.001 | 1.10 (0.62-1.96) | 0.75 | 1.54 (0.85-2.78) | 0.15 |
| 6 | 4.85 (2.79-8.43) | <0.001 | 1.29 (0.72-2.33) | 0.40 | 1.78 (0.97-3.27) | 0.06 |
| 7 or more | 6.01 (3.39-10.67) | <0.001 | 1.43 (0.78-2.64) | 0.25 | 2.04 (1.08-3.85) | 0.03 |

Model 1: age, initial NIHSS, prior anticoagulation

Mode 2: age, initial NIHSS, BMI, arrival time, dyslipidemia, smoking, prior anticoagulation, prior statin, antihypertensive treatment, antidiabetic treatment, statin treatment, reperfusion therapy, creatinine, glucose, SBP, large artery diseases, multiple lesions, stroke mechanism (TOAST)

Supplemental Table VI. Association of CHA2DS2-VASc scores with vascular events in the no OAC treatment group (n=1895)

| (A) Primary outcome | Crude HR (95% CI) | P | Model 1 (HR, 95% CI) | P | Model 2 (HR, 95% CI) | P |
| --- | --- | --- | --- | --- | --- | --- |
| Per 1-point increase | 1.13 (1.08-1.18) | <0.001 | 1.02 (0.97-1.07) | 0.53 | 1.03 (0.97-1.08) | 0.34 |
| 0 | ref |  | ref |  | ref |  |
| 1 | 1.23 (0.65-2.32) | 0.52 | 1.03 (0.55-1.94) | 0.93 | 1.09 (0.58-2.07) | 0.78 |
| 2 | 1.91 (1.07-3.43) | 0.03 | 1.10 (0.61-1.99) | 0.76 | 1.19 (0.66-2.16) | 0.56 |
| 3 | 2.34 (1.33-4.12) | 0.003 | 1.13 (0.63-2.02) | 0.69 | 1.28 (0.71-2.31) | 0.41 |
| 4 | 2.33 (1.33-4.10) | 0.003 | 0.96 (0.53-1.73) | 0.90 | 1.16 (0.64-2.11) | 0.62 |
| 5 | 2.40 (1.36-4.26) | 0.003 | 1.06 (0.59-1.92) | 0.84 | 1.19 (0.65-2.17) | 0.57 |
| 6 | 2.77 (1.53-4.99) | 0.001 | 1.12 (0.61-2.08) | 0.71 | 1.36 (0.72-2.54) | 0.34 |
| 7 or more | 3.22 (1.73-5.98) | 0.0002 | 1.27 (0.67-2.42) | 0.47 | 1.39 (0.72-2.69) | 0.33 |
| (B) Recurrent stroke |  |  |  |  |  |  |
| Per 1-point increase | 1.14 (1.04-1.25) | 0.01 | 1.17 (1.06-1.29) | 0.002 | 1.15 (1.04-1.28) | 0.01 |
| 0 | ref |  | ref |  | ref |  |
| 1 | 2.19 (0.48-10.01) | 0.31 | 2.31 (0.51-10.55) | 0.28 | 2.17 (0.47-9.93) | 0.32 |
| 2 | 3.24 (0.76-13.80) | 0.11 | 3.94 (0.92-16.91) | 0.06 | 3.93 (0.91-16.95) | 0.07 |
| 3 | 3.33 (0.80-13.83) | 0.10 | 4.28 (1.01-18.10) | 0.048 | 4.15 (0.97-17.69) | 0.05 |
| 4 | 2.95 (0.71-12.29) | 0.14 | 4.03 (0.94-17.21) | 0.06 | 3.96 (0.92-17.12) | 0.07 |
| 5 | 3.11 (0.74-13.14) | 0.12 | 4.15 (0.96-17.98) | 0.06 | 4.07 (0.92-17.90) | 0.06 |
| 6 | 4.67 (1.09-20.05) | 0.04 | 6.39 (1.44-28.34) | 0.01 | 5.86 (1.30-26.49) | 0.02 |
| 7 or more | 5.69 (1.27-25.43) | 0.02 | 7.98 (1.73-36.86) | 0.01 | 6.94 (1.47-32.85) | 0.01 |
| (C) All-cause mortality |  |  |  |  |  |  |
| Per 1-point increase | 1.15 (1.10-1.21) | <0.001 | 1.00 (0.94-1.06) | 0.98 | 1.02 (0.97-1.08) | 0.44 |
| 0 | ref |  | ref |  | ref |  |
| 1 | 1.06 (0.52-2.13) | 0.88 | 0.80 (0.40-1.62) | 0.54 | 0.88 (0.43-1.78) | 0.72 |
| 2 | 1.77 (0.93-3.35) | 0.08 | 0.78 (0.41-1.50) | 0.46 | 0.85 (0.44-1.63) | 0.62 |
| 3 | 2.40 (1.30-4.44) | 0.01 | 0.82 (0.43-1.54) | 0.53 | 0.97 (0.51-1.84) | 0.92 |
| 4 | 2.41 (1.31-4.45) | 0.01 | 0.66 (0.35-1.26) | 0.21 | 0.86 (0.45-1.64) | 0.64 |
| 5 | 2.37 (1.27-4.41) | 0.01 | 0.73 (0.38-1.40) | 0.35 | 0.87 (0.45-1.69) | 0.69 |
| 6 | 2.90 (1.53-5.49) | 0.001 | 0.80 (0.41-1.56) | 0.51 | 1.05 (0.53-2.09) | 0.88 |
| 7 or more | 3.31 (1.69-6.48) | 0.001 | 0.89 (0.44-1.79) | 0.74 | 1.06 (0.52-2.17) | 0.88 |

Model 1: age, initial NIHSS, prior anticoagulation

Mode 2: age, initial NIHSS, BMI, arrival time, dyslipidemia, smoking, prior anticoagulation, prior statin, antihypertensive treatment, antidiabetic treatment, statin treatment, reperfusion therapy, creatinine, glucose, SBP, large artery diseases, multiple lesions, stroke mechanism (TOAST)

Supplemental Table VII. Comparisons of OAC vs OAC+AP in anticoagulated patients with stroke and AF (N=5500)

|  | OAC | OAC+AP | P | ASD |
| --- | --- | --- | --- | --- |
| N | 4440 | 1060 |  |  |
| Age, mean (SD) | 72.7±10.2 | 72.7±9.8 | 0.95 | 0.0021 |
| Male, n (%) | 2410 (54.3) | 605 (57.1) | 0.10 | 0.0563 |
| Arrival time, n (%) |  |  | 0.003 | 0.1114 |
| within 12 h | 3597 (81.0) | 812 (76.6) |  |  |
| 12-24 h | 477 (10.7) | 130 (12.3) |  |  |
| 24-48 h | 366 (8.2) | 118 (11.1) |  |  |
| Prestroke mRS 0-1, n (%) | 3845 (86.6) | 920 (86.8) | 0.87 | 0.0057 |
| BMI, mean (SD) | 23.6±3.5 | 23.6±3.4 | 0.91 | 0.0037 |
| Baseline NIHSS, median (IQR) | 5 (2 - 9) | 4 (2 - 9) | 0.18 | 0.0455 |
| Risk factors, n (%) |  |  |  |  |
| Previous TIA | 99 (2.2) | 23 (2.2) | 0.91 | 0.0041 |
| Previous stroke | 1032 (23.2) | 318 (30.0) | <0.001 | 0.1533 |
| Previous PAD | 19 (0.4) | 17 (1.6) | <0.001 | 0.1175 |
| Previous CAD | 459 (10.3) | 304 (28.7) | <0.001 | 0.4758 |
| Hypertension | 3013 (67.9) | 818 (77.2) | <0.001 | 0.2097 |
| Diabetes | 1145 (25.8) | 389 (36.7) | <0.001 | 0.2370 |
| Dyslipidemia | 1264 (28.5) | 333 (31.4) | 0.06 | 0.0644 |
| Smoking | 777 (17.5) | 219 (20.7) | 0.02 | 0.0805 |
| Congestive heart failure | 202 (4.5) | 40 (3.8) | 0.27 | 0.0389 |
| Medication history |  |  |  |  |
| Prior antiplatelet use | 1438 (32.4) | 507 (47.8) | <0.001 | 0.3191 |
| Prior anticoagulant use | 854 (19.2) | 204 (19.2) | 0.99 | 0.0003 |
| Prior antihypertensive use | 2583 (58.2) | 728 (68.7) | <0.001 | 0.2194 |
| Prior statin use | 977 (22.0) | 339 (32.0) | <0.001 | 0.2262 |
| Prior antidiabetic use | 849 (19.1) | 298 (28.1) | <0.001 | 0.2129 |
| Laboratory findings, mean (SD) |  |  |  |  |
| WBC count, 10^3^/µL | 7.94±2.70 | 7.96±2.61 | 0.77 | 0.0099 |
| Creatinine, mg/dl | 1.03±0.88 | 1.08±0.86 | 0.14 | 0.0513 |
| Hemoglobin, g/dl | 13.5±1.9 | 13.6±1.9 | 0.77 | 0.0101 |
| Platelet count, 10^3^/µL | 206.4±62.0 | 211.5±64.9 | 0.02 | 0.0804 |
| Low-density lipoprotein, mg/dl | 98.6±32.1 | 93.9±32.1 | <0.001 | 0.1464 |
| PT, INR | 1.16±0.39 | 1.19±0.43 | 0.10 | 0.0581 |
| Glucose, mg/dl | 135.3±49.9 | 142.6±58.4 | 0.0002 | 0.1359 |
| SBP, mmHg | 142.7±25.0 | 142.1±25.7 | 0.45 | 0.0254 |
| LAD, n (%) |  |  | <0.001 | 0.2400 |
| no stenosis | 2072 (46.7) | 451 (42.5) |  |  |
| mild <50% | 211 (4.8) | 80 (7.5) |  |  |
| moderate >50% | 375 (8.4) | 153 (14.4) |  |  |
| occlusion | 1782 (40.1) | 376 (35.5) |  |  |
| Multiple lesions, n (%) | 698 (15.7) | 173 (16.3) | 0.63 | 0.0164 |
| Recanalization therapy |  |  | <0.001 | 0.1894 |
| no | 3174 (71.5) | 835 (78.8) |  |  |
| IVT | 692 (15.6) | 118 (11.1) |  |  |
| IAT | 274 (6.2) | 54 (5.1) |  |  |
| IV+IAT | 300 (6.8) | 53 (5.0) |  |  |
| CHA2DS2-VASc, med (IQR) | 3 (2 - 4) | 4 (3 - 5) | <0.001 | 0.2835 |
| Stroke mechanism |  |  | <0.001 | 0.3028 |
| other than CE | 743 (16.7) | 311 (29.3) |  |  |
| CE | 3697 (83.3) | 749 (70.7) |  |  |
| In-hospital treatment |  |  |  |  |
| Antidiabetics | 841 (18.9) | 273 (25.8) | <0.001 | 0.1641 |
| Anti-HTN | 2045 (46.1) | 553 (52.2) | 0.0003 | 0.1225 |
| Statin | 3754 (84.5) | 937 (88.4) | 0.002 | 0.1127 |

^†^ P-value by Pearson chi-square test, Student's t-test or Wilcoxon rank sum test as appropriate

*An ASD of >0.1 is considered a meaningful imbalance.

Supplemental Table VIII. Propensity score analysis

|  | IPTW | | |
| --- | --- | --- | --- |
|  | OAC | OAC+AP | ASD |
| N | 5499 | 5540 |  |
| Age, mean (SD) | 72.7 (11.3) | 72.7 (23.2) | 0.002 |
| Male, n (%) | 3004 (54.6) | 2953 (53.3) | 0.03 |
| Arrival time, n (%) |  |  | <0.001 |
| Within 12 h | 4414 (80.3) | 4440 (80.1) |  |
| 12-24 h | 602 (10.9) | 591 (10.7) |  |
| 24-48 h | 483 (8.8) | 509 (9.2) |  |
| Pre mRS 0-1, n (%) | 4764 (86.6) | 4766 (86.0) | 0.02 |
| BMI, mean (SD) | 23.6 (3.9) | 23.4 (7.8) | 0.02 |
| NIHSS, median (IQR) | 5 (2 - 9) | 5 (2 - 10) | 0.02 |
| Risk factors, n (%) |  |  |  |
| Previous TIA | 122 (2.2) | 104 (1.9) | 0.02 |
| Previous stroke | 1344 (24.4) | 1332 (24.0) | 0.01 |
| Previous PAD | 39 (0.7) | 35 (0.6) | 0.01 |
| Previous CAD | 761 (13.8) | 742 (13.4) | 0.01 |
| Hypertension | 3825 (69.6) | 3826 (69.1) | 0.01 |
| Diabetes | 1534 (27.9) | 1539 (27.8) | 0.003 |
| Dyslipidemia | 1590 (28.9) | 1634 (29.5) | 0.01 |
| Smoking | 999 (18.2) | 987 (17.8) | 0.01 |
| Congestive heart failure | 243 (4.4) | 254 (4.6) | 0.01 |
| Medication history |  |  |  |
| Prior antiplatelet | 1949 (35.4) | 2035 (36.7) | 0.03 |
| Prior anticoagulant | 1051 (19.1) | 978 (17.7) | 0.04 |
| Prior antihypertensive use | 3306 (60.1) | 3271 (59.0) | 0.02 |
| Prior statin use | 1315 (23.9) | 1286 (23.2) | 0.02 |
| Prior antidiabetic use | 1145 (20.8) | 1120 (20.2) | 0.02 |
| Laboratory findings, mean (SD) |  |  |  |
| WBC count, 10^3^/µL | 7.94 (3.00) | 8.01 (6.06) | 0.02 |
| Creatinine, mg/dl | 1.04 (0.99) | 1.02 (1.60) | 0.01 |
| Hemoglobin, g/dl | 13.5 (2.1) | 13.6 (4.4) | 0.01 |
| Platelet count, 10^3^/µL | 207.2 (69.6) | 207.3 (139.2) | 0.001 |
| Low-density lipoprotein, mg/dl | 97.8 (35.8) | 99.1 (76.5) | 0.02 |
| PT, INR | 1.17 (0.45) | 1.16 (0.86) | 0.01 |
| Glucose, mg/dl | 136.8 (57.4) | 137.1 (118.8) | 0.003 |
| SBP, mmHg | 142.6 (27.9) | 142.5 (58.4) | 0.004 |
| LAD, n (%) |  |  | 0.04 |
| no stenosis | 2513 (45.7) | 2411 (43.5) |  |
| mild <50% | 287 (5.2) | 279 (5.0) |  |
| moderate >50% | 535 (9.7) | 560 (10.1) |  |
| occlusion | 2164 (39.4) | 2291 (41.3) |  |
| Multiple lesions, n (%) | 867 (15.8) | 888 (16.0) | 0.01 |
| Recanalization therapy |  |  | 0.03 |
| no | 4004 (72.8) | 3975 (71.7) |  |
| IVT | 812 (14.8) | 861 (15.5) |  |
| IAT | 328 (6.0) | 332 (6.0) |  |
| IV+IAT | 355 (6.5) | 372 (6.7) |  |
| Mechanism |  |  | 0.0003 |
| other than CE | 1054 (19.2) | 1062 (19.2) |  |
| CE | 4445 (80.8) | 4478 (80.8) |  |
| In-hospital treatment |  |  |  |
| Antidiabetics | 1110 (20.2) | 1111 (20.1) | 0.003 |
| Anti-HTN | 2588 (47.1) | 2525 (45.6) | 0.03 |
| Statin | 4686 (85.2) | 4654 (84.0) | 0.03 |

Supplemental Table IX. Vascular events of OAC vs OAC+AP according to the CHA2DS2-VASc score subgroups

|  | Crude | | | IPTW | | |
| --- | --- | --- | --- | --- | --- | --- |
|  | OAC | OAC+AP | P-value^a^ | OAC | OAC+AP | P-value^b^ |
| N |  |  |  |  |  |  |
| Primary outcome | 1-year event rate,  % (95% CI) | 1-year event rate,  % (95% CI) |  | Weighted event rate,  % (95% CI) | Weighted event rate,  % (95% CI) |  |
| All anticoagulated | 10.06 (9.13-10.99) | 15.64 (13.36-17.91) | <.0001 | 10.35 (9.52-11.17) | 16.92 (15.89-17.93) | <.0001 |
| Low score subgroup | 4.47 (3.35-5.58) | 11.00 (6.89-15.11) | 0.0001 | 4.54 (3.52-5.56) | 14.92 (13.15-16.66) | <.0001 |
| Intermediate score subgroup | 10.35 (8.93-11.78) | 14.91 (11.42-18.40) | 0.0082 | 10.09 (8.84-11.33) | 15.31 (13.81-16.77) | 0.0064 |
| High score subgroup | 17.13 (14.75-19.51) | 19.43 (15.31-23.55) | 0.2316 | 17.60 (15.57-19.59) | 21.88 (19.62-24.08) | 0.1018 |
| All recurrent stroke |  |  |  |  |  |  |
| All anticoagulated | 3.73 (3.14-4.32) | 6.97 (5.35-8.59) | <.0001 | 4.02 (3.48-4.56) | 7.27 (6.55-7.99) | 0.0002 |
| Low score subgroup | 1.95 (1.20-2.69) | 5.36 (2.39-8.33) | 0.0045 | 2.01 (1.32-2.70) | 6.16 (4.94-7.36) | 0.0016 |
| Intermediate score subgroup | 3.71 (2.82-4.60) | 6.28 (3.89-8.67) | 0.0297 | 3.80 (3.01-4.59) | 6.29 (5.28-7.29) | 0.0458 |
| High score subgroup | 6.30 (4.73-7.87) | 8.85 (5.78-11.92) | 0.1051 | 6.82 (5.43-8.18) | 10.36 (8.63-12.05) | 0.0543 |
| All-cause mortality |  |  |  |  |  |  |
| All anticoagulated | 7.29 (6.48-8.11) | 11.34 (9.31-13.36) | <.0001 | 7.32 (6.60-8.03) | 12.89 (11.96-13.82) | <.0001 |
| Low score subgroup | 2.64 (1.76-3.52) | 6.85 (3.47-10.23) | 0.0019 | 2.66 (1.86-3.45) | 10.92 (9.35-12.46) | <.0001 |
| Intermediate score subgroup | 7.83 (6.56-9.11) | 11.44 (8.25-14.63) | 0.0206 | 7.50 (6.38-8.59) | 12.12 (10.75-13.48) | 0.0071 |
| High score subgroup | 12.73 (10.59-14.88) | 14.10 (10.43-17.78) | 0.4396 | 12.68 (10.87-14.45) | 16.36 (14.30-18.37) | 0.1192 |
| Hemorrhagic stroke |  |  |  |  |  |  |
| All anticoagulated | 0.21 (0.06-0.35) | 0.63 (0.07-1.19) | 0.1050 | 0.24 (0.09-0.39) | 0.49 (0.27-0.70) | 0.3441 |
| Low score subgroup | 0.31 (0.01-0.62) | 0.48 (0.00-1.41) | 0.7090 | 0.30 (0.02-0.58) | 0.26 (0.00-0.53) | 0.9330 |
| Intermediate score subgroup | 0.19 (0.00-0.40) | 0.86 (0.00-1.83) | 0.0890 | 0.22 (0.02-0.43) | 0.42 (0.13-0.70) | 0.5999 |
| High score subgroup | 0.10 (0.00-0.29) | 0.48 (0.00-1.43) | 0.4276 | 0.15 (0.00-0.40) | 1.00 (0.30-1.70) | 0.0502 |

^a^ P-value by log-rank test

^b^ P-value by IPTW log-rank test

Supplemental Table X. Association of OAC vs OAC+AP with other vascular outcomes according to the CHA2DS2-VASc score subgroup

|  | Crude HR | p | Pint | Cox PH* | p | Pint | IPTWb | p | Pint |
| --- | --- | --- | --- | --- | --- | --- | --- | --- | --- |
| (A) Recurrent stroke |  |  |  |  |  |  |  |  |  |
| all anticoagulated |  |  |  |  |  |  |  |  |  |
| OAC | ref |  |  | ref |  |  | ref |  |  |
| OAC+AP | 1.84 (1.38-2.44) | <0.001 | 0.36 | 1.57 (1.17-2.10) | 0.003 | 0.27 | 1.84 (1.31-2.58) | 0.0004 | 0.32 |
| low score |  |  |  |  |  |  |  |  |  |
| OAC | ref |  |  | ref |  |  | ref |  |  |
| OAC+AP | 2.57 (1.30-5.05) | 0.01 |  | 2.59 (1.31-5.11) | 0.01 |  | 3.13 (1.40-6.99) | 0.01 |  |
| intermediate score |  |  |  |  |  |  |  |  |  |
| OAC | ref |  |  | ref |  |  | ref |  |  |
| OAC+AP | 1.65 (1.04-2.60) | 0.03 |  | 1.57 (0.99-2.49) | 0.06 |  | 1.67 (0.99-2.83) | 0.05 |  |
| high score |  |  |  |  |  |  |  |  |  |
| OAC | ref |  |  | ref |  |  | ref |  |  |
| OAC+AP | 1.43 (0.93-2.20) | 0.11 |  | 1.33 (0.86-2.06) | 0.20 |  | 1.55 (0.94-2.54) | 0.08 |  |
| (B) All-cause mortality |  |  |  |  |  |  |  |  |  |
| all anticoagulated |  |  |  |  |  |  |  |  |  |
| OAC | ref |  |  | ref |  |  | ref |  |  |
| OAC+AP | 1.60 (1.29-1.99) | <0.001 | 0.07 | 1.56 (1.24-1.95) | 0.0001 | 0.04 | 1.82 (1.38-2.40) | <0.001 | 0.02 |
| low score |  |  |  |  |  |  |  |  |  |
| OAC | ref |  |  | ref |  |  | ref |  |  |
| OAC+AP | 2.55 (1.39-4.65) | 0.002 |  | 3.07 (1.67-5.63) | 0.0003 |  | 4.38 (2.03-9.48) | 0.0002 |  |
| intermediate score |  |  |  |  |  |  |  |  |  |
| OAC | ref |  |  | ref |  |  | ref |  |  |
| OAC+AP | 1.49 (1.06-2.08) | 0.02 |  | 1.66 (1.18-2.33) | 0.004 |  | 1.67 (1.15-2.44) | 0.01 |  |
| high score |  |  |  |  |  |  |  |  |  |
| OAC | ref |  |  | ref |  |  | ref |  |  |
| OAC+AP | 1.15 (0.83-1.60) | 0.40 |  | 1.28 (0.92-1.79) | 0.14 |  | 1.32 (0.91-1.92) | 0.15 |  |

*Adjusted variables: age, NIHSS scores, dyslipidemia, smoking, prior anticoagulant use, prior statin use, prior antihypertensive use, prior antidiabetic use, LAD, glucose, SBP, LDL cholesterol, multiple lesions, reperfusion therapy, stroke mechanism, CHA2DS2-VASc score.

^b^ Weighted Cox proportional hazards model with robust standard errors
